# Influence of Supraliminal Reward Information on Unconsciously Triggered Response Inhibition

**DOI:** 10.1371/journal.pone.0108530

**Published:** 2014-09-30

**Authors:** Liuting Diao, Cody Ding, Senqing Qi, Qinghong Zeng, Bo Huang, Mengsi Xu, Lingxia Fan, Dong Yang

**Affiliations:** 1 Key Laboratory of Cognition and Personality (Southwest University), Ministry of Education, Chongqing, China; 2 Center for Mental Health Research, Southwest University, Chongqing, China; 3 University of Missouri, St. Louis, Missouri, United States of America; 4 MOE Key Laboratory of Modern Teaching Technology, Shaanxi Normal University, Xi'an, China; Duke University Medical Center, United States of America

## Abstract

Although executive functions (e.g., response inhibition) are often thought to interact consciously with reward, recent studies have demonstrated that they can also be triggered by unconscious stimuli. Further research has suggested a close relationship between consciously and unconsciously triggered response inhibition. To date, however, the effect of reward on unconsciously triggered response inhibition has not been explored. To address this issue, participants in this study performed runs of a modified Go/No-Go task during which they were exposed to both high and low value monetary rewards presented both supraliminally and subliminally. Participants were informed that they would earn the reward displayed if they responded correctly to each trial of the run. According to the results, when rewards were presented supraliminally, a greater unconsciously triggered response inhibition was observed for high-value rewards than for low-value rewards. In contrast, when rewards were presented subliminally, no enhanced unconsciously triggered response inhibition was observed. Results revealed that supraliminal and subliminal rewards have distinct effects on unconsciously triggered response inhibition. These findings have important implications for extending our understanding of the relationship between reward and response inhibition.

## Introduction

A considerable amount of research has demonstrated that people invest an increased amount of effort in tasks when rewards are at stake, regardless of whether such rewards are consciously perceived [1]–[3]. However, there is ongoing debate as to whether supraliminal and subliminal reward information influence executive functions in similar ways. On one hand, several studies have shown that both supraliminal and subliminal reward information have similar effects on executive functions with high-value rewards enhancing task performance [4]–[6]. On the other hand, numerous recent studies have also concluded that supraliminal and subliminal reward information have distinct effects on task performance [7]–[10]. Specifically, supraliminal, not subliminal, reward information can influence executive functions. Traditional views hold that high-level cognitive control functions require attention and consciousness [11], [12]. Recent studies, however, have shown that response inhibition, a key component of high-level executive control functions, can be triggered unconsciously [13]–[15]. For example, van Gaal and colleagues [17] instructed participants to perform a modified Go/No-Go task that included weakly masked No-Go trials, strongly masked No-Go trials, as well as Go trials. Interestingly, results showed that the strongly masked No-Go trials elongated ongoing task behavior compared with the strongly masked Go trials, suggesting that unconsciously perceived stimuli triggered response inhibition. The study further revealed that unconsciously triggered response inhibition was strongly associated with the pre-supplementary motor area and the inferior frontal cortex, which relates to the same network employed in conscious response inhibition [16]. Subsequent studies have reported similar neural mechanisms for both consciously and unconsciously triggered response inhibition [15], [17], [18] despite a limited amount of research suggesting the presence of dissociable mechanisms [19]. It seems plausible that there is a close relationship between consciously and unconsciously triggered response inhibition.

To our knowledge, comparably little research has investigated whether reward can influence unconsciously triggered executive functions (e.g., unconsciously triggered response inhibition). In the present study, therefore, we attempted to examine this question using a modified Go/No-Go task in combination with the reward-priming paradigm [3]. In the reward-priming task, participants were supraliminally or subliminally exposed to a low-value or high-value reward they could earn by performing well on a modified Go/No-Go task. Through this approach, we sought to investigate how performance-contingent supraliminal and subliminal reward information influence unconsciously triggered response inhibition.

Building on previous studies exploring conscious (supraliminal) and unconscious (subliminal) information processes [20]–[23], we hypothesized that supraliminal and subliminal rewards would influence unconsciously triggered response inhibition in distinct ways. First, we hypothesized that participants would experience greater unconsciously triggered response inhibition for supraliminal high-value rewards than for low-value rewards. Given that the neural activation magnitude of an unconscious inhibition network could predict the unconsciously initialed RT slowing (i.e. mean RT of strongly masked No-Go trials minus strongly masked Go trials; for review, see van Gaal et al. [17]) and correlated positively with it, we hypothesized that participants would induce greater unconsciously triggered response inhibition for supraliminal high-value rewards, as shown by larger amount of RT slowing. Second, we hypothesized that high-value rewards would not significantly boost task performance when presented subliminally.

## Materials and Methods

### Ethics statement

The ethics committee of Southwest University of China approved this experiment. Written informed consent was obtained from all participants in compliance with the principles contained in the Declaration of Helsinki.

### Participants

A total of 35 undergraduates (20 women, 15 men; age range  = 19–24 years; mean age  = 21.76, SD = 1.76) from Southwest University in China participated in our study. All participants were right-handed with normal or corrected-to-normal vision. Upon completion of the trials, they received any money earned during the experiment. Data from one participant were excluded from the analysis due to an above-chance discrimination of the primes.

### Stimuli and procedure

Stimuli were displayed on a 20-inch Dell monitor (Dell, Inc., Round Rock, Texas) with a 60 Hz refresh rate. Participants viewed the display from a distance of about 70 cm so that each centimeter subtended a visual angle of 0.82^°^. The E-Prime software (Psychology Software Tools, Inc., Sharpsburg, PA) was used for stimulus presentation and behavioral data collection.

**Figure 1:**
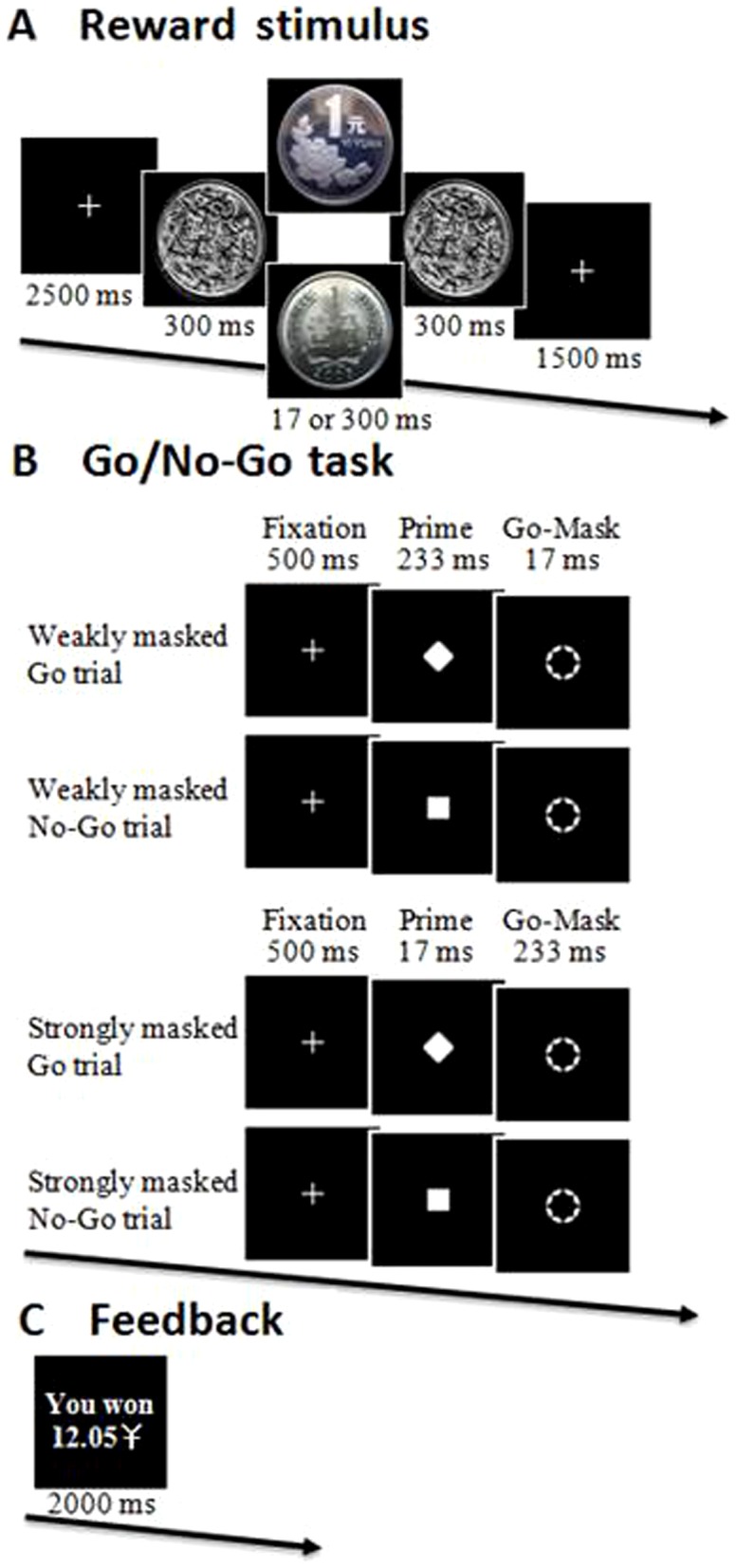
Experimental design. Participants were informed that, if they responded correctly to each of the 32 trials in a Go/No-Go task (B), they would receive the reward that was displayed at the beginning of the run (A). Participants were instructed that cumulative earnings would appear at the end of each run (C). Each run included 16 weakly masked trials and 16 strongly masked trials.

Participants performed 48 runs during the experiment. At the beginning of each run, a fixation cross appeared (2500 ms) followed sequentially by a pre-mask (300 ms), the reward stimulus (17 or 300 ms), a post-mask (300 ms), another fixation cross (1500 ms, see Figure 1A), and a modified Go/No-Go task of 32 trials (see Figure 1B). Participants were informed that they would receive the reward presented at the beginning of the run if they responded correctly to each of the 32 trials. The cumulative earnings attained were presented at the end of each run (see Figure 1C). Participants were instructed that the reward stimuli were either 1 cent or 1 yuan (approximately 100 cents) and that sometimes they would be difficult to perceive.

The modified Go/No-Go task was adapted from van Gaal et al. [17] and consisted of 32 trials made up of eight of each of the four trial types (weakly masked Go trials, weakly masked No-Go trials, strongly masked Go trials, and strongly masked No-Go trials). For the weakly masked conditions, a fixation point (500 ms) appeared, followed by a Go or No-Go prime for a relatively long duration (233 ms) and a briefly presented annulus (17 ms). In the strongly masked conditions, the presentation duration of prime and annulus were 17 ms and 233 ms, respectively. The purpose of the strongly masked condition in the experimental design was to ensure that the annulus acted as a metacontrast mask, since this works effectively in reducing stimulus visibility [24]. Thus, in the strongly masked condition, participants were incapable of perceiving the Go or No-Go signals and just perceived a white annulus. Duration was equal for all trials (750 ms), and all trials were presented in random order. The between-trial interval was 1000–1500 ms.

Participants were instructed to respond to a white annulus (visual angle of 0.8^°^) as quickly as possible by pressing the “*m*” key on a standard keyboard with their right index finger but to withhold their response when a white square (visual angle of 0.47^°^×0.47^°^) preceded the annulus. However, participants were instructed to respond to a white diamond (the same square revolved by 45^°^) preceding the annulus as quickly as possible by pressing the “*m*” key with their right index finger in a standard keyboard. The No-Go signal stimulus (diamond or square) was counterbalanced among participants.

To detect whether the modified Go/No-Go task induced unconsciously triggered response inhibition, 21 participants first performed the task in a pilot study, and the data of the strongly masked Go and No-Go trials were analyzed. The results of this pilot demonstrated that participants showed slower response when responding to the strongly masked No-Go trials (*M* = 389.75 ms, *SD* = 44.33) than in the strongly masked Go trials (*M* = 382.78 ms, *SD* = 43.53; *t* [20]  = 2.92, *p* = .008), indicating that unconsciously triggered response inhibition occurred during the modified Go/No-Go task. A two-choice discrimination test suggested that the primes (diamond and square) could not be perceived under the strongly masked conditions (mean percentage correct  = 49.6%, *SD* = 0.03; *d′* = 0.15, *SD* = 0.43, *t* [20]  = 1.55, *p* = .14).

Each participant completed a training run before performing the formal 48-run experiment. After completion of the last run, each participant underwent two tests aimed at detecting whether he or she could perceive the reward and prime stimuli that were presented subliminally. First, subjects completed a forced-choice test focused on the reward stimuli. Each trial consisted of a reward stimulus and two mask stimuli identical to those in the main experiment, followed by four choices on a black screen instead of the Go/No-Go task. Participants were asked to press the “1” key if they were sure that they saw “1 cent,” the “2” key if they were sure that they saw “1 yuan,” the “3” key if they thought they had probably seen “1 cent,” and the “4” key if they thought they had probably see “1 yuan.” The test was comprised of 96 trials that included 24 trials of each of the four reward conditions (supraliminally presented 1 cent, supraliminally presented 1 yuan, subliminally presented 1 cent, and subliminally presented 1 yuan). In this task, the importance of accuracy rather than speed was emphasized to the participants. The choices remained on the screen until the participants made a response.

The second test assessed whether participants could discriminate between the strongly masked No-Go trials and the strongly masked Go trials in a two-alternative forced-choice test. In this test, participants performed four blocks of 32 trials (16 trials of each strongly masked condition) with the same stimuli and procedure as was used for the main experiment. A choice-selection screen followed each trial. Participants were instructed that they should press the “*v*” key if they saw the No-Go signal (the diamond) and the “*n*” key if they saw the Go signal (the square). Before performing this test, participants were told that diamonds and squares would be presented with equal frequency. The importance of accuracy over speed was similarly emphasized in this task. The choices remained on the screen until the participants selected a response.

### Data analysis

The percentage of correct runs was analyzed with a 2×2 repeated measures ANOVA with reward value (1 cent and 1 yuan) and reward presentation duration (17 ms and 300 ms) as within-subjects factors. Only correct responses in which a reward could be earned were analyzed. RTs less than 100 or greater than 1000 were excluded from the analysis [16]. Mean RTs were entered into a 2×2×2 repeated measures ANOVA with reward value (1 cent and 1 yuan), reward presentation duration (17 ms and 300 ms), and trial type (strongly masked Go trial and strongly masked No-Go trial) as within-subjects factors. RT slowing was analyzed in a 2×2 repeated measures ANOVA with reward value (1 cent and 1 yuan) and reward presentation duration (17 ms and 300 ms) as within-subjects factors. Detection performance (percentage correct) was tested for each participant using a binominal test (*p*<.05). At the group level, a one-sample *t* test was performed on the *d* scores (test against zero).

## Results

### Reward and prime visibility test

In the forced-choice test measuring monetary reward visibility, participants perceived 98.9% (*SD* = 0.02) of rewards when presented supraliminally, indicating that participants could perceive the value of the reward. All of the participants reported that they could not consciously perceive the subliminally presented rewards, and the mean percentages of correct responses did not differ significantly from chance level (mean percentage of correct responses  = 50.86%, *SD* = 0.08, *p* = .54). Furthermore, *d* scores did not differ significantly from zero (*d′* = 0.05, *SD* = 1.87, *t*[33]  = 0.16, *p* = .87). These results indicate that participants could perceive rewards when presented supraliminally but not when presented subliminally.

In the two-alternative forced-choice test that measured Go and No-Go prime visibility, all of the participants reported that they could not consciously perceive the strongly masked signals, the mean percentage of correct responses did not differ significantly from chance level (mean percentage of correct responses  = 49.7%, *SD* = 0.07, *p* = .82), and *d* scores did not differ significantly from zero (*d′* = 0.08, *SD* = 1.37, *t*[33]  = 0.34, *p* = .73). These results showed that participants could not perceive the Go and No-Go prime in the strongly masked condition.

### Percentage of correct runs

**Figure 2:**
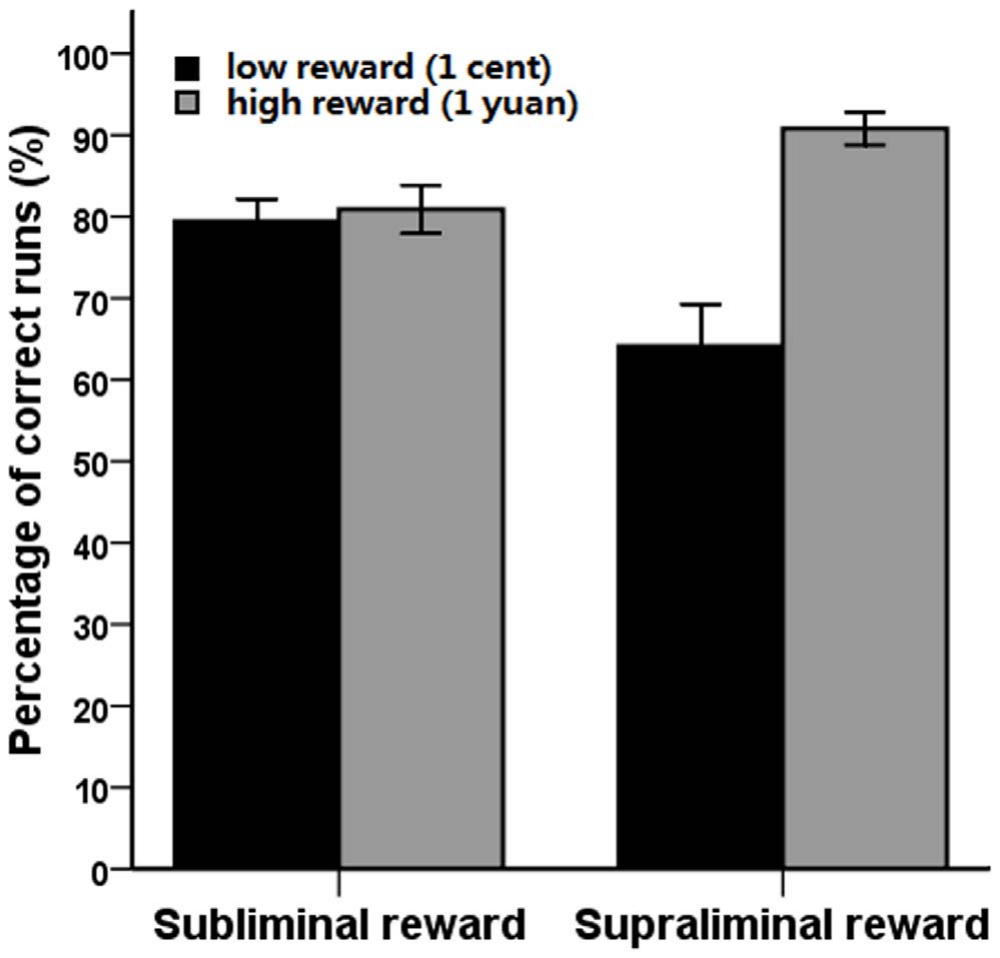
The percentage of correct runs as a function of reward value and reward presentation duration. High rewards and low rewards have no significant difference in effect on percentage of correct run when presented subliminally (left panel). High rewards elicited higher percentage of correct runs than low rewards when presented supraliminally (right panel). Error bars represent standard errors of the mean.

Analysis of the percentage of correct runs indicated a significant main effect of reward value (*F*[1, 33]  = 22.78, *p*<.001) interacting with reward presentation duration (*F*[1, 33]  = 23.33, *p*<.001). Further analysis revealed that, when the rewards were presented supraliminally, participants had a higher percentage of correct runs when they had the possibility of earning the high-value rewards (*M* = 90.81%, *SD* = 0.12) compared to the low-value rewards (*M* = 64.09%, *SD* = 0.30; *F*[1, 33]  = 29.98, *p*<.001). This implies that being conscious of the potential of a high-value reward instigated greater effort among the participants than did the low-value rewards. This effect was not observed, however, when rewards were presented subliminally (1 yuan, *M* = 80.88%, *SD* = 0.17; 1 cent, *M* = 79.41%, *SD* = 0.16; *F*<1; see Figure 2). No other effect was observed within the percentage of correct runs.

### Reaction times

ANOVA yielded a significant main effect of trial type (*F*[1, 33]  = 109.73, *p*<.001) with faster responses recorded for the strongly masked Go trials (*M* = 390.86 ms, *SD* = 49.15) than for the No-Go trials (*M* = 404.79 ms, *SD* = 47.74). This finding was consistent with previous findings indicating that unconsciously triggered response inhibition existed under reward conditions.

Further analysis of RTs showed that reward value had a significant main effect (*F*[1, 33]  = 18.56, *p* = .002), which interacted with reward presentation duration (*F*[1, 33]  = 26.95, *p*<.001). Participants also recorded a slower response when given the opportunity to earn 1 yuan (*M* = 334.82 ms, *SD* = 48.92) compared with 1 cent (*M* = 321.22 ms, *SD* = 50.02) in the supraliminal conditions (*F*[1,33]  = 29.33, *p*<.001) but not in the subliminal conditions (1 yuan, *M* = 328.78 ms, *SD* = 47.85; 1 cent, *M* = 333.84 ms, *SD* = 48.84; *F*<1). These findings suggest that participants slowed their responses when conscious of the potential of earning high-value rewards.

**Figure 3:**
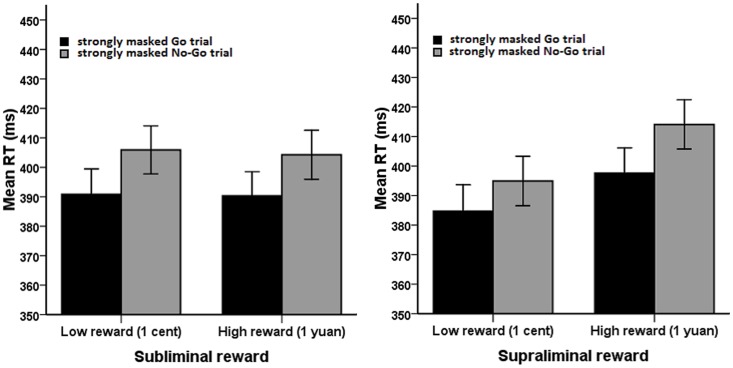
Mean RTs of trial type as a function of reward value and reward presentation duration. (A) High rewards and low rewards have no significant difference in effect on either mean RT of strongly masked Go trials or mean RT of strongly masked No-Go trials when presented subliminally. (B) High rewards elicited slower mean RT both on strongly masked Go trials and on strongly masked No-Go trials than did low rewards when presented supraliminally. Error bars represent standard errors of the mean.

Importantly, we found an interaction effect among the three experimental factors (*F*[1, 33]  = 6.19, *p* = .018). To interpret this three-way interaction, we conducted a 2 (reward value) ×2 (trial type) repeated measures ANOVA separately for the two reward presentation durations (subliminal and supraliminal conditions). In the subliminal condition (reward duration  = 17 ms), only trial type had a significant main effect (*F*[1, 33]  = 105.98, *p*<.001) in which participants responded faster for strongly masked Go trials (*M* = 390.56 ms, *SD* = 48.75) than for No-Go trials (*M* = 405.08 ms, *SD* = 47.82; see Figure 3A). Under supraliminal conditions (reward duration  = 300 ms), both reward value (*F*[1,33]  = 29.33, *p*<.001) and trial type (*F*[1,33]  = 69.65, *p*<.001) had significant main effects, and the interaction between reward value and trial type was also significant (*F*[1, 33]  = 5.58, *p* = .024; see Figure 3B). Further analysis revealed that strongly masked Go trials resulted in slower responses for 1 yuan (*M* = 397.60 ms, *SD* = 49.87) than for 1 cent (*M* = 384.71 ms, *SD* = 51.96; *F*[1,33]  = 13.48, *p* = .001) and that slower responses were also found for strongly masked No-Go trials with high-value rewards (*M* = 414.08 ms, *SD* = 48.44) than for those with low-value rewards (*M* = 394.92 ms, *SD* = 49.04; *F*[1, 33]  = 42.27, *p*<.001). No other effect was observed for RTs.

### Reaction time slowing

**Figure 4:**
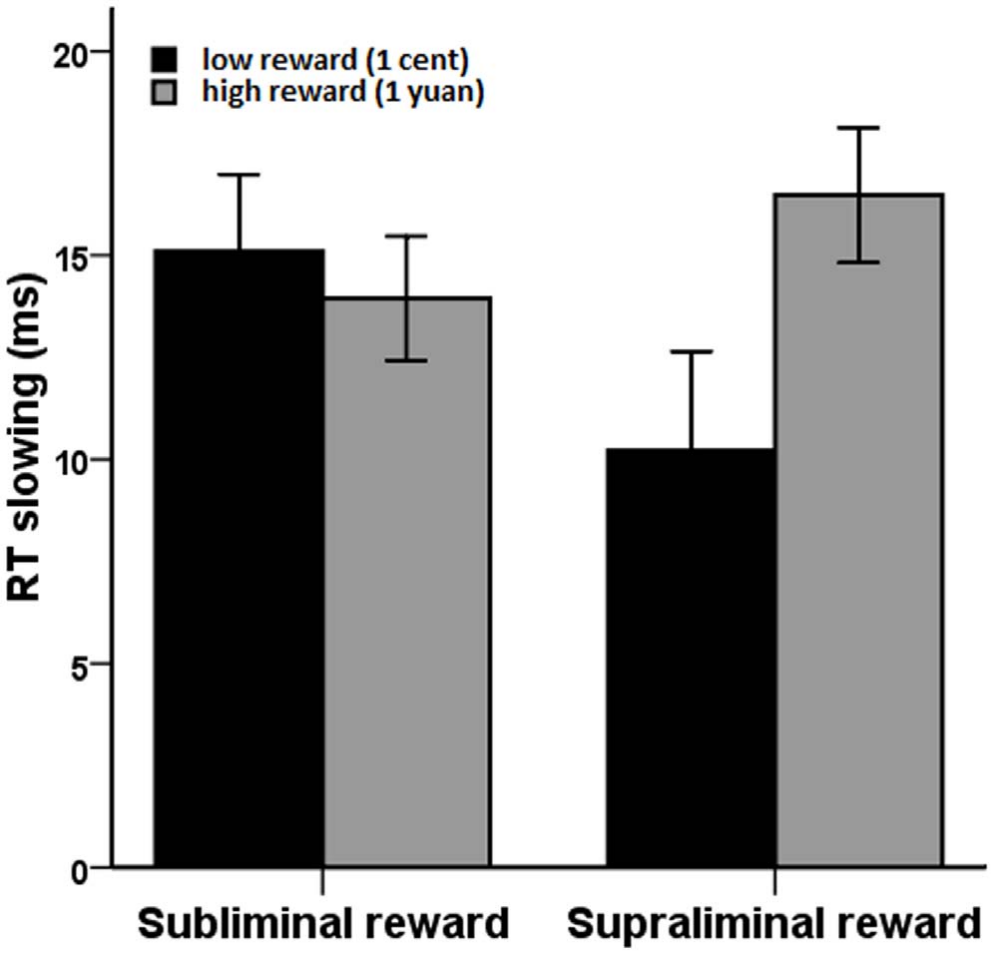
RT slowing as a function of reward value and reward presentation duration. High rewards and low rewards have no significant difference in effect on RT slowing when presented subliminally (left panel). High rewards elicited larger amount of RT slowing than low rewards when presented supraliminally (right panel). Error bars represent standard errors of the mean.

Importantly, analysis of RT slowing revealed that reward values significantly interacted with reward presentation duration (*F*[1,33]  = 6.19, *p* = .018). Follow-up analysis showed that RT slowing for high-value rewards (*M* = 16.47 ms, *SD* = 9.63) was significantly more than for low-value rewards (*M* = 10.22 ms, *SD* = 14.15) when rewards were presented supraliminally (*F*[1,33]  = 5.58, *p* = .024). These findings suggest that being conscious of high-value rewards enhanced unconsciously triggered response inhibition. However, no effect was observed of high-value rewards on improved task performance when rewards were presented subliminally (1 yuan, *M* = 13.94 ms, *SD* = 8.85; 1 cent, *M* = 15.09 ms, *SD* = 11.00; *F*<1; see Figure 4). No other effect was observed for RT slowing.

## Discussion

In this study, we combined the reward-priming paradigm and a modified Go/No-Go task to investigate the effects of supraliminal and subliminal reward information on unconsciously triggered response inhibition. Results showed that participants induced greater unconsciously triggered response inhibition for high-value rewards than for low-value rewards when presented supraliminally. This may suggest that being conscious of the notion of supraliminal high-value rewards enhances unconsciously triggered response inhibition. However, this enhanced performance was not observed when rewards were presented subliminally, lending support to the notion that supraliminal and subliminal rewards have distinct effects on the unconscious information process.

Participants increased unconsciously triggered response inhibition for high-value rewards when presented supraliminally, which is consistent with previous research suggesting that supraliminal (conscious) high-value rewards can enhance executive functions [7], [8]. These findings could be interpreted in three ways. First, previous studies have demonstrated that being conscious of high-value reward enables individuals to increase their effort and level of engagement in cognitive tasks, resulting in better performance. In this study, participants were shown to employ more effort for supraliminal high-value rewards, as reflected by higher percentage of correct runs. Second, our findings are in line with the theory that conscious awareness of rewards enables participants to employ strategic behavior [9] on attainment of high-value rewards and to actively prevent the waste of mental resources on attainment of low-value rewards. Third, our findings may also be interpreted in terms of the neural mechanisms that underlie the processing of supraliminal reward information and unconsciously triggered response inhibition. Several studies have revealed that the prefrontal cortical network plays a crucial role in relation to supraliminal rewards information [25]–[27]. Interestingly, prior work has demonstrated that the mechanisms associated with unconsciously triggered response inhibition are located mainly in the prefrontal cortical network [16]. We speculate, therefore, that, when a participant is consciously aware of high-value rewards, his or her prefrontal cortex is more active in the process, resulting in greater unconsciously triggered response inhibition.

However, this effect was not observed when rewards were presented subliminally. In the forced-choice test on reward visibility, we observed that participants could not distinguish the value of coins when presented subliminally, suggesting that participants could not perceive the value of subliminally presented rewards. Further, in this study, participants were not shown to recruit strategies to attain a higher percentage of correct runs for subliminal high-value rewards, suggesting that participants might not employ more effort for subliminal high-value rewards. We speculate, therefore, that subliminal high-value rewards might disable recruit strategies to invest more effort in processing the task performance, resulting in the lack of reward effect. In line with previous theoretical frameworks [20], [28], our findings revealed that, in the complex modified Go/No-Go task, subliminal reward processing is limited when attempting to recruit strategies to enhance task performance.

Similar to previous studies [7]–[9], our study confirmed that supraliminal and subliminal rewards have distinct effects on executive functions. These findings converged well with the framework provided by Bijleveld et al. [5], which separates reward processing into two stages: initial (unconscious/subliminal) and full (conscious/supraliminal) reward processing. Full reward processing is required in order for the brain to develop strategies to affect behavior. This processing type may involve higher-level cognitive functions located in the prefrontal cortex that are related to unconscious inhibition control [13], [16]. In contrast, the initial reward processing that underlies executive functions is accompanied by activity in rudimentary brain structures (e.g., the ventral striatum) that rarely correlate with unconscious inhibition control [3], [16], [29]. This would explain why supraliminal reward processing could influence task performance when subliminal reward processing could not.

The present findings have both theoretical and practical implications. They expand our understanding of the relationship between reward and executive function as it has been demonstrated that conscious rewards can influence not only consciously but also unconsciously triggered executive function. Additionally, several studies have used enhanced (un)conscious response inhibition to change habitual behavior that may be detrimental to health, such as alcohol abuse [30], [31]. As our findings suggest that supraliminal reward information can improve unconscious response inhibition, this may provide an effective method to benefit individuals who abuse alcohol.

Several limitations of the present study should be noted. One limitation is that our study did not consider influence of personality. Bustin and colleagues suggested that personality (e.g., novelty seeking) can affect the impact of rewards on executive function [32]. Future research should take this factor into consideration. Second, we did not consider the impact of intrinsic motivation (e.g., interest) in this experiment. Although we successfully induced participants' extrinsic motivation through monetary reward incentives, we cannot completely rule out the effect of intrinsic motivation on task performance. Future research should attempt to replicate the present findings while controlling for the impact of intrinsic motivation.

In conclusion, the present study first revealed that supraliminal high-value rewards could enhance unconsciously triggered response inhibition when subliminal high-value reward could not. Our findings, therefore, lend support to the notion that supraliminal and subliminal rewards have distinct effects on unconsciously triggered response inhibition. Our findings provide insight into the relationship between reward and response inhibition.

## Supporting Information

Table S1 **Data of the preliminary test.**
(DOC)

Table S2 **Date of the percentage of correct runs in the formal experiment.**
(DOC)

Table S3 **Mean Reaction time and RT slowing in the formal experiment.**
(DOC)

Table S4 **Data of reward and prime visibility in the formal experiment.**
(DOC)

## References

[1] Aarts H, Custers R, Marien H (2008) Preparing and motivating behavior outside of awareness. Science 319: 1639. 18356517 10.1126/science.1150432

[2] Capa RL, Cleeremans A, Bustin GM, Hansenne M (2011) Long-lasting effect of subliminal processes on cardiovascular responses and performance. Int J Psychophysiol 81: 22–30. 21515314 10.1016/j.ijpsycho.2011.04.001

[3] Pessiglione M, Schmidt L, Draganski B, Kalisch R, Lau H, et al (2007) How the brain translates money into force: a neuroimaging study of subliminal motivation. Science 316: 904–906. 17431137 10.1126/science.1140459 PMC2631941

[4] Bijleveld E, Custers R, Aarts H (2009) The unconscious eye opener: pupil dilation reveals strategic recruitment of resources upon presentation of subliminal reward cues. Psychol Sci 20: 1313–1315. 19788532 10.1111/j.1467-9280.2009.02443.x

[5] Bijleveld E, Custers R, Aarts H (2012) Adaptive Reward Pursuit: How Effort Requirements Affect Unconscious Reward Responses and Conscious Reward Decisions. Journal of Experimental Psychology-General 141: 728–742. 22468672 10.1037/a0027615

[6] Capa RL, Bustin GM, Cleeremans A, Hansenne M (2011) Conscious and Unconscious Reward Cues Can Affect a Critical Component of Executive Control (Un)conscious Updating? Exp Psychol 58: 370–375. 21310696 10.1027/1618-3169/a000104

[7] Bijleveld E, Custers R, Aarts H (2010) Unconscious reward cues increase invested effort, but do not change speed-accuracy tradeoffs. Cognition 115: 330–335. 20089247 10.1016/j.cognition.2009.12.012

[8] Capa RL, Bouquet CA, Dreher JC, Dufour A (2013) Long-lasting effects of performance-contingent unconscious and conscious reward incentives during cued task-switching. Cortex 49: 1943–1954. 22770561 10.1016/j.cortex.2012.05.018

[9] Zedelius CM, Veling H, Aarts H (2012) When unconscious rewards boost cognitive task performance inefficiently: the role of consciousness in integrating value and attainability information. Front Hum Neurosci 6: 219. 22848198 10.3389/fnhum.2012.00219 PMC3404454

[10] Zedelius CM, Veling H, Bijleveld E, Aarts H (2012) Promising high monetary rewards for future task performance increases intermediate task performance. PLoS ONE 7: e42547. 22905145 10.1371/journal.pone.0042547 PMC3414454

[11] Jack A, Shallice T (2001) Introspective physicalism as an approach to the science of consciousness. Cognition 79: 161–196. 11164027 10.1016/s0010-0277(00)00128-1

[12] Norman D, Shallice T (1986) Attention to action: willed and automatic control of behavior. In: Consciousness and self-regulation (Davidson RJ, Schwartz GE, Shapiro D, eds). New York: Plenum.

[13] van Gaal S, Ridderinkhof KR, Fahrenfort JJ, Scholte HS, Lamme VA (2008) Frontal cortex mediates unconsciously triggered inhibitory control. J Neurosci 28: 8053–8062. 18685030 10.1523/JNEUROSCI.1278-08.2008 PMC6670759

[14] van Gaal S, Lamme VA, Ridderinkhof KR (2010) Unconsciously triggered conflict adaptation. PLoS ONE 5: e11508. 20634898 10.1371/journal.pone.0011508 PMC2901348

[15] Wokke ME, van Gaal S, Scholte HS, Ridderinkhof KR, Lamme VA (2011) The flexible nature of unconscious cognition. PLoS ONE 6: e25729. 21980530 10.1371/journal.pone.0025729 PMC3182242

[16] van Gaal S, Ridderinkhof KR, Scholte HS, Lamme VA (2010) Unconscious activation of the prefrontal no-go network. J Neurosci 30: 4143–4150. 20237284 10.1523/JNEUROSCI.2992-09.2010 PMC6632275

[17] Lenartowicz A, Verbruggen F, Logan GD, Poldrack RA (2011) Inhibition-related activation in the right inferior frontal gyrus in the absence of inhibitory cues. J Cogn Neurosci 23: 3388–3399. 21452946 10.1162/jocn_a_00031

[18] van Gaal S, de Lange FP, Cohen MX (2012) The role of consciousness in cognitive control and decision making. Front Hum Neurosci 6: 121. 22586386 10.3389/fnhum.2012.00121 PMC3345871

[19] van Gaal S, Lamme VA, Fahrenfort JJ, Ridderinkhof KR (2011) Dissociable brain mechanisms underlying the conscious and unconscious control of behavior. J Cogn Neurosci 23: 91–105. 20175675 10.1162/jocn.2010.21431

[20] Bijleveld E, Custers R, Aarts H (2012) Human reward pursuit: From rudimentary to higher-level functions. Current Directions in Psychological Science 21: 273–273.

[21] Dehaene S, Changeux JP, Naccache L, Sackur J, Sergent C (2006) Conscious, preconscious, and subliminal processing: a testable taxonomy. Trends Cogn Sci 10: 204–211. 16603406 10.1016/j.tics.2006.03.007

[22] Dehaene S, Kerszberg M, Changeux JP (1998) A neuronal model of a global workspace in effortful cognitive tasks. Proc Natl Acad Sci U S A 95: 14529–14534. 9826734 10.1073/pnas.95.24.14529 PMC24407

[23] Van den Bussche E, Hughes G, Humbeeck NV, Reynvoet B (2010) The relation between consciousness and attention: an empirical study using the priming paradigm. Conscious Cogn 19: 86–97. 20097581 10.1016/j.concog.2009.12.019

[24] Breitmeyer B, Hoar W, Randall D, Conte F (1984) Visual masking: An integrative approach: Clarendon Press Oxford.

[25] Jimura K, Locke HS, Braver TS (2010) Prefrontal cortex mediation of cognitive enhancement in rewarding motivational contexts. Proc Natl Acad Sci U S A 107: 8871–8876. 20421489 10.1073/pnas.1002007107 PMC2889311

[26] Kouneiher F, Charron S, Koechlin E (2009) Motivation and cognitive control in the human prefrontal cortex. Nat Neurosci 12: 939–945. 19503087 10.1038/nn.2321

[27] Miller EK, Cohen JD (2001) An integrative theory of prefrontal cortex function. Annu Rev Neurosci 24: 167–202. 11283309 10.1146/annurev.neuro.24.1.167

[28] Zedelius CM, Veling H, Custers R, Bijleveld E, Chiew KS, et al (2014) A new perspective on human reward research: How consciously and unconsciously perceived reward information influences performance. Cogn Affect Behav Neurosci: 1–16. 10.3758/s13415-013-0241-z 24399682

[29] Pessiglione M, Petrovic P, Daunizeau J, Palminteri S, Dolan RJ, et al (2008) Subliminal instrumental conditioning demonstrated in the human brain. Neuron 59: 561–567. 18760693 10.1016/j.neuron.2008.07.005 PMC2572733

[30] Houben K, Nederkoorn C, Wiers RW, Jansen A (2011) Resisting temptation: decreasing alcohol-related affect and drinking behavior by training response inhibition. Drug Alcohol Depend 116: 132–136. 21288663 10.1016/j.drugalcdep.2010.12.011

[31] Jones A, Field M (2013) The effects of cue-specific inhibition training on alcohol consumption in heavy social drinkers. Exp Clin Psychopharmacol 21: 8–16. 23181512 10.1037/a0030683

[32] Bustin GM, Quoidbach J, Hansenne M, Capa RL (2012) Personality modulation of (un)conscious processing: Novelty Seeking and performance following supraliminal and subliminal reward cues. Conscious Cogn 21: 947–952. 22475476 10.1016/j.concog.2012.03.005

