# Supplementary material for: Influence of Supraliminal Reward Information on Unconsciously Triggered Response Inhibition
Source: PLoS One. 2014 Sep 30;9(9):e108530. doi: 10.1371/journal.pone.0108530 (PMC4182473; doi:10.1371/journal.pone.0108530)
Supplement: Table S1 — Data of the preliminary test. (DOC) [file pone.0108530.s001.doc]

| ID | RT of go (ms) | RT of no-go (ms) | Correct percentage (%) | Hit rate | Z of hit rate | False rate | Z of false rate | d score |
| --- | --- | --- | --- | --- | --- | --- | --- | --- |
| 1 | 398.37 | 434.97 | 0.49 | 0.75 | 0.68 | 0.76 | 0.71 | -0.03 |
| 2 | 330.66 | 346.62 | 0.5 | 0.57 | 0.18 | 0.56 | 0.15 | 0.03 |
| 3 | 377.27 | 378.04 | 0.49 | 0.67 | 0.44 | 0.69 | 0.5 | -0.06 |
| 4 | 405.85 | 420.94 | 0.49 | 0.5 | 0 | 0.52 | 0.05 | -0.05 |
| 5 | 338.44 | 351.58 | 0.5 | 0.62 | 0.31 | 0.62 | 0.31 | 0 |
| 6 | 391 | 400.32 | 0.51 | 0.63 | 0.33 | 0.61 | 0.28 | 0.05 |
| 7 | 339.38 | 341.38 | 0.5 | 0.99 | 2.33 | 0.99 | 2.33 | 0 |
| 8 | 337.12 | 343.34 | 0.51 | 0.99 | 2.33 | 0.99 | 2.33 | 0 |
| 9 | 441.94 | 447.05 | 0.53 | 0.38 | 1.18 | 0.33 | 0.96 | 0.22 |
| 10 | 364.74 | 365.15 | 0.51 | 0.56 | 0.15 | 0.54 | 0.1 | 0.05 |
| 11 | 361.44 | 363.99 | 0.49 | 0.64 | 0.36 | 0.66 | 0.41 | -0.05 |
| 12 | 405.37 | 428.68 | 0.48 | 0.57 | 0.18 | 0.61 | 0.28 | -0.1 |
| 13 | 392.11 | 394.23 | 0.52 | 0.59 | 0.23 | 0.54 | 0.1 | 0.13 |
| 14 | 465.48 | 475.47 | 0.56 | 0.63 | 0.33 | 0.51 | 0.03 | 0.3 |
| 15 | 357.3 | 368.38 | 0.44 | 0.41 | 1.34 | 0.54 | 0.1 | 1.24 |
| 16 | 428.39 | 431.53 | 0.48 | 0.67 | 0.44 | 0.71 | 0.56 | -0.12 |
| 17 | 380.53 | 395 | 0.48 | 0.66 | 0.41 | 0.7 | 0.53 | -0.12 |
| 18 | 317.46 | 317.23 | 0.49 | 0.59 | 0.23 | 0.6 | 0.26 | -0.02 |
| 19 | 462.45 | 449.62 | 0.45 | 0.45 | 1.65 | 0.55 | 0.13 | 1.52 |
| 20 | 332.51 | 329.85 | 0.54 | 0.41 | 1.34 | 0.33 | 0.96 | 0.39 |
| 21 | 410.61 | 401.37 | 0.45 | 0.61 | 0.28 | 0.71 | 0.56 | -0.28 |

Table_S1. Data of the preliminary test.

Note:

“ID” means “identification of participants”;

“RT of go” means “Mean reaction time of strongly masked go trial”;

“RT of no-go” means “Mean reaction time of strongly masked no-go trial”;

"Correct percentage" means "mean correct response percentage”;

"Hit rate" means "hit rate";

"Z of hit rate" means "Z score of hit rate";

"False rate" means "false rate";

"Z of false rate" means "Z score of false rate";

"d score" is equal to "Z score of hit rate minus Z score of false rate".
