# Supplementary material for: Influence of Supraliminal Reward Information on Unconsciously Triggered Response Inhibition
Source: PLoS One. 2014 Sep 30;9(9):e108530. doi: 10.1371/journal.pone.0108530 (PMC4182473; doi:10.1371/journal.pone.0108530)
Supplement: Table S2 — Date of the percentage of correct runs in the formal experiment. (DOC) [file pone.0108530.s002.doc]

Table_S2. Date of the percentage of correct runs in the formal experiment.

| ID | gender | age | sub_1 | sup_1 | sub_100 | sup_100 |
| --- | --- | --- | --- | --- | --- | --- |
| 1 | 2 | 23 | 0.75 | 0.58 | 0.92 | 0.92 |
| 2 | 1 | 20 | 0.5 | 0.58 | 0.67 | 1 |
| 3 | 1 | 24 | 1 | 1 | 1 | 1 |
| 4 | 2 | 22 | 0.92 | 0.67 | 1 | 1 |
| 5 | 2 | 23 | 0.92 | 0.67 | 1 | 1 |
| 6 | 2 | 19 | 0.58 | 1 | 0.83 | 1 |
| 7 | 1 | 24 | 0.83 | 1 | 0.75 | 1 |
| 8 | 1 | 22 | 1 | 1 | 1 | 1 |
| 9 | 1 | 21 | 1 | 1 | 1 | 0.92 |
| 10 | 2 | 21 | 0.92 | 0.42 | 0.67 | 1 |
| 11 | 1 | 23 | 1 | 1 | 1 | 1 |
| 12 | 1 | 20 | 0.92 | 0.58 | 0.83 | 0.92 |
| 13 | 2 | 20 | 0.92 | 0.67 | 1 | 0.83 |
| 14 | 2 | 22 | 0.67 | 0.17 | 0.83 | 0.92 |
| 15 | 1 | 24 | 0.92 | 0.92 | 0.92 | 0.92 |
| 16 | 1 | 23 | 0.92 | 1 | 0.92 | 1 |
| 17 | 1 | 21 | 0.58 | 0.25 | 0.42 | 0.83 |
| 18 | 2 | 22 | 0.92 | 0.83 | 0.92 | 0.92 |
| 19 | 1 | 22 | 0.92 | 0.67 | 0.58 | 0.83 |
| 20 | 1 | 24 | 0.75 | 0.88 | 0.88 | 1 |
| 21 | 2 | 19 | 0.69 | 0.63 | 0.63 | 0.56 |
| 22 | 2 | 19 | 0.75 | 0.5 | 0.75 | 0.88 |
| 23 | 2 | 23 | 0.56 | 0.88 | 0.75 | 0.69 |
| 24 | 1 | 18 | 0.83 | 0.25 | 0.67 | 0.92 |
| 25 | 1 | 22 | 0.67 | 0.17 | 0.83 | 0.83 |
| 26 | 2 | 24 | 0.92 | 0.42 | 0.58 | 0.67 |
| 27 | 1 | 23 | 0.58 | 0.5 | 0.5 | 0.67 |
| 28 | 1 | 21 | 0.75 | 0.58 | 0.75 | 1 |
| 29 | 1 | 21 | 0.5 | 0.08 | 0.83 | 0.83 |
| 30 | 1 | 21 | 0.83 | 0.17 | 0.92 | 0.83 |
| 31 | 1 | 22 | 0.58 | 0.67 | 0.42 | 1 |
| 32 | 2 | 24 | 1 | 1 | 1 | 1 |
| 33 | 2 | 19 | 0.67 | 0.17 | 0.92 | 1 |
| 34 | 2 | 24 | 0.75 | 0.92 | 0.83 | 1 |

Note:

“ID” means “identification of participants”;

“gender”: “1” means ”female”; “2” means ”male”;

“sub_1” means “percentage of correct runs in subliminal 1 cent condition”

“sub_100” means “percentage of correct runs in subliminal 1 yuan condition”

“sup_1” means “percentage of correct runs in supraliminal 1 cent condition”

“sup_100” means “percentage of correct runs in supraliminal 1 yuan condition”
