# Supplementary material for: Influence of Supraliminal Reward Information on Unconsciously Triggered Response Inhibition
Source: PLoS One. 2014 Sep 30;9(9):e108530. doi: 10.1371/journal.pone.0108530 (PMC4182473; doi:10.1371/journal.pone.0108530)
Supplement: Table S3 — Mean Reaction time and RT slowing in the formal experiment. (DOC) [file pone.0108530.s003.doc]

Table_S3. Mean Reaction time and RT slowing in the formal experiment.

| ID | sub_1_  go (ms) | sub_1_  no-go  (ms) | sup_100_  go  (ms) | sup_100_  no-go  (ms) | sup_1_  go  (ms) | sup_1_  no-go  (ms) | sup_100_  go  (ms) | sup_100_  no-go  (ms) | RTS of sub_1  (ms) | RTS of  sub_100  (ms) | RTS of sup_1  (ms) | RTS of  sup_100  (ms) |
| --- | --- | --- | --- | --- | --- | --- | --- | --- | --- | --- | --- | --- |
| 1 | 335.46 | 357.62 | 344.52 | 349.98 | 357.66 | 362.44 | 360.82 | 367.82 | 22.16 | 4.78 | 5.46 | 7.00 |
| 2 | 339.24 | 356.26 | 340.81 | 361.66 | 342.69 | 360.66 | 353.27 | 370.5 | 17.02 | 17.97 | 20.85 | 17.23 |
| 3 | 525.48 | 527.66 | 514.14 | 521.98 | 537.21 | 553.2 | 535.51 | 540.93 | 2.18 | 15.99 | 7.84 | 5.42 |
| 4 | 396.45 | 406.61 | 373.41 | 383.49 | 381.35 | 393.28 | 411.97 | 415.45 | 10.16 | 11.93 | 10.08 | 3.48 |
| 5 | 364.94 | 383.01 | 374.79 | 376.94 | 367.82 | 379.06 | 377.06 | 397.7 | 18.07 | 11.24 | 2.15 | 20.64 |
| 6 | 397.35 | 403.15 | 394.33 | 384.26 | 384.24 | 396.25 | 387.79 | 406.55 | 5.8 | 12.01 | -10.07 | 18.76 |
| 7 | 326.97 | 348.43 | 332.63 | 339.85 | 331.3 | 344 | 337.47 | 361.11 | 21.46 | 12.7 | 7.22 | 23.64 |
| 8 | 390.28 | 412.84 | 356.30 | 380.18 | 381.7 | 404.8 | 393.85 | 412.69 | 22.56 | 23.1 | 23.88 | 18.84 |
| 9 | 434.77 | 454.18 | 424.18 | 445.86 | 417.63 | 442.86 | 432.07 | 449.65 | 19.41 | 25.23 | 21.68 | 17.58 |
| 10 | 361.65 | 368.66 | 351.38 | 360.32 | 370.9 | 380.69 | 367.81 | 383.75 | 7.01 | 9.79 | 8.94 | 15.94 |
| 11 | 407.43 | 431.49 | 417.92 | 436.63 | 423.8 | 442.13 | 432.36 | 448.1 | 24.06 | 18.33 | 18.71 | 15.74 |
| 12 | 346.45 | 372.01 | 361.45 | 367.68 | 363.53 | 359.17 | 353.69 | 364.63 | 25.56 | -4.36 | 6.23 | 10.94 |
| 13 | 346.49 | 359.03 | 351.03 | 350.92 | 364.95 | 365.64 | 364.85 | 371.65 | 12.54 | 0.69 | -0.11 | 6.80 |
| 14 | 370.1 | 378.57 | 357.14 | 346.86 | 377.83 | 383.00 | 380.71 | 390.85 | 8.47 | 5.17 | -10.28 | 10.14 |
| 15 | 404.66 | 430.67 | 398.54 | 423.18 | 406.78 | 435.55 | 405.8 | 426.92 | 26.01 | 28.77 | 24.64 | 21.12 |
| 16 | 435.64 | 447.26 | 426.82 | 421.00 | 421.92 | 437.65 | 423.63 | 441.83 | 11.62 | 15.73 | -5.82 | 18.2 |
| 17 | 407.62 | 426.68 | 385.07 | 406.75 | 394.66 | 419.87 | 406.97 | 436.58 | 19.06 | 25.21 | 21.68 | 29.61 |
| 18 | 423.31 | 403.33 | 427.50 | 403.14 | 391.40 | 398.88 | 392.91 | 428.91 | -19.98 | 7.48 | -24.36 | 36.00 |
| 19 | 429.46 | 431.14 | 383.34 | 406.79 | 422.30 | 416.67 | 444.34 | 457.07 | 1.68 | -5.63 | 23.45 | 12.73 |
| 20 | 400.90 | 416.14 | 403.06 | 406.30 | 403.04 | 411.63 | 402.44 | 414.21 | 15.24 | 8.59 | 3.24 | 11.77 |
| 21 | 420.53 | 451.50 | 435.41 | 442.47 | 426.31 | 449.81 | 429.32 | 459.56 | 30.97 | 23.5 | 7.06 | 30.24 |
| 22 | 536.68 | 546.24 | 539.94 | 534.60 | 522.64 | 530.70 | 530.05 | 539.47 | 9.56 | 8.06 | -5.34 | 9.42 |
| 23 | 473.40 | 478.45 | 469.31 | 489.49 | 467.18 | 474.05 | 487.77 | 498.99 | 5.05 | 6.87 | 20.18 | 11.22 |
| 24 | 389.99 | 406.31 | 404.62 | 409.10 | 388.49 | 398.64 | 391.03 | 402.08 | 16.32 | 10.15 | 4.48 | 11.05 |
| 25 | 347.20 | 356.86 | 359.83 | 371.38 | 346.93 | 366.87 | 334.11 | 347.14 | 9.66 | 19.94 | 11.55 | 13.03 |
| 26 | 378.41 | 421.98 | 373.86 | 403.01 | 389.68 | 411.66 | 381.23 | 425.77 | 43.57 | 21.98 | 29.15 | 44.54 |
| 27 | 327.53 | 340.16 | 314.70 | 327.73 | 322.90 | 334.67 | 335.48 | 352.20 | 12.63 | 11.77 | 13.03 | 16.72 |
| 28 | 363.24 | 375.31 | 336.84 | 364.94 | 372.09 | 380.00 | 362.84 | 391.96 | 12.07 | 7.91 | 28.1 | 29.12 |
| 29 | 352.48 | 374.07 | 310.57 | 338.57 | 345.44 | 358.79 | 360.86 | 365.18 | 21.59 | 13.35 | 28 | 4.32 |
| 30 | 330.37 | 355.10 | 305.42 | 344.95 | 321.89 | 347.78 | 319.63 | 350.00 | 24.73 | 25.89 | 39.53 | 30.37 |
| 31 | 394.07 | 394.85 | 383.82 | 400.67 | 376.57 | 403.23 | 407.36 | 413.02 | 0.78 | 26.66 | 16.85 | 5.66 |
| 32 | 373.19 | 395.06 | 376.49 | 372.50 | 381.33 | 408.54 | 398.81 | 406.76 | 21.87 | 27.21 | -3.99 | 7.95 |
| 33 | 403.01 | 415.56 | 390.20 | 377.88 | 409.19 | 424.26 | 438.55 | 450.07 | 12.55 | 15.07 | -12.32 | 11.52 |
| 34 | 352.76 | 374.54 | 360.64 | 376.39 | 357.02 | 368.06 | 376.20 | 389.52 | 21.78 | 11.04 | 15.75 | 13.32 |

Note:

“sub_1_go” means “mean RT of strongly masked go trials in subliminal 1 cent condition”;

“sub_1_no-go” means “mean RT of strongly masked no-go trials in subliminal 1 cent condition”;

“sub_100_go” means “mean RT of strongly masked go trials in subliminal 1 yuan condition”;

“sub_100_no-go” means “mean RT of strongly masked no-go trials in subliminal 1 cent condition”;

“sup_1_go” means “mean RT of strongly masked go trials in supraliminal 1 cent condition”;

“sup_1_no-go” means “mean RT of strongly masked no-go trials in supraliminal 1 cent condition”;

“sup_100_go” means “mean RT of strongly masked go trials in supraliminal 1 yuan condition”;

“sup_100_no-go” means “mean RT of strongly masked no-go trials in supraliminal 1 yuan condition”;

“RTS” means “RT slowing(Mean RT of strongly masked no-go trials minus strongly masked go trials)”

“RTS of sub_1” means “RT slowing of subliminal 1 cent condition”;

“RTS of sub_100” means “RT slowing of subliminal 1 yuan condition”;

“RTS of sup_1” means “RT slowing of supraliminal 1 cent condition”;

“RTS of sup_100” means “RT slowing of supraliminal 1 yuan condition”;
