# Supplementary material for: Influence of Supraliminal Reward Information on Unconsciously Triggered Response Inhibition
Source: PLoS One. 2014 Sep 30;9(9):e108530. doi: 10.1371/journal.pone.0108530 (PMC4182473; doi:10.1371/journal.pone.0108530)
Supplement: Table S4 — Data of reward and prime visibility in the formal experiment. (DOC) [file pone.0108530.s004.doc]

Table_S4. Data of reward and prime visibility in the formal experiment.

| ID | data of reward visibility | | | | | | data of prime visibility | | | | | |
| --- | --- | --- | --- | --- | --- | --- | --- | --- | --- | --- | --- | --- |
|  | correct  percent-  age | hit rate | Z of hit rate | false rate | Z of false rate | d score | mean correct | hit rate | Z of hit rate | false rate | Z of false rate | d score |
| 1 | 0.46 | 0.42 | 1.41 | 0.50 | 4.00 | -2.59 | 0.54 | 0.48 | 2.05 | 0.40 | 1.28 | 0.77 |
| 2 | 0.50 | 0.50 | 4.00 | 0.50 | 4.00 | 0 | 0.47 | 0.48 | 2.05 | 0.54 | 1.10 | 0.95 |
| 3 | 0.67 | 0.67 | 0.44 | 0.33 | 0.96 | -0.51 | 0.52 | 0.94 | 1.56 | 0.90 | 1.28 | 0.28 |
| 4 | 0.54 | 0.67 | 0.44 | 0.58 | 0.20 | 0.24 | 0.39 | 0.42 | 1.41 | 0.64 | 0.36 | 1.05 |
| 5 | 0.46 | 0.33 | 0.96 | 0.42 | 1.41 | -0.45 | 0.47 | 0.64 | 0.36 | 0.70 | 0.53 | -0.17 |
| 6 | 0.42 | 0.25 | 0.68 | 0.42 | 1.41 | -0.73 | 0.37 | 0.74 | 0.64 | 1.00 | 4.00 | -3.36 |
| 7 | 0.50 | 1.00 | 4.00 | 1.00 | 4.00 | 0 | 0.51 | 0.48 | 2.05 | 0.46 | 1.75 | 0.30 |
| 8 | 0.63 | 0.50 | 4.00 | 0.25 | 0.68 | 3.33 | 0.43 | 0.40 | 1.28 | 0.54 | 0.10 | 1.18 |
| 9 | 0.33 | 0.33 | 0.96 | 0.67 | 0.44 | 0.51 | 0.51 | 0.58 | 0.20 | 0.56 | 0.15 | 0.05 |
| 10 | 0.54 | 0.50 | 4.00 | 0.42 | 1.41 | 2.59 | 0.53 | 0.70 | 0.53 | 0.64 | 0.36 | 0.17 |
| 11 | 0.50 | 0.42 | 1.41 | 0.42 | 1.41 | 0 | 0.46 | 0.46 | 1.75 | 0.54 | 0.10 | 1.65 |
| 12 | 0.50 | 0.57 | 0.18 | 0.57 | 0.18 | 0 | 0.38 | 0.38 | 1.18 | 0.62 | 0.31 | 0.87 |
| 13 | 0.42 | 0.33 | 0.96 | 0.50 | 4.00 | -3.05 | 0.50 | 0.56 | 0.15 | 0.56 | 0.15 | 0 |
| 14 | 0.42 | 0.33 | 0.96 | 0.50 | 4.00 | -3.05 | 0.52 | 0.58 | 0.2 | 0.54 | 0.10 | 0.10 |
| 15 | 0.43 | 0.36 | 1.08 | 0.50 | 4.00 | -2.92 | 0.47 | 0.38 | 1.18 | 0.44 | 1.56 | -0.38 |
| 16 | 0.50 | 0.42 | 1.41 | 0.42 | 1.41 | 0 | 0.49 | 0.54 | 1.10 | 0.56 | 0.15 | 0.95 |
| 17 | 0.50 | 0.42 | 1.41 | 0.42 | 1.41 | 0 | 0.52 | 0.56 | 1.15 | 0.52 | 0.05 | 1.1 |
| 18 | 0.58 | 0.67 | 0.44 | 0.50 | 4.00 | -3.56 | 0.67 | 0.84 | 0.99 | 0.5 | 4.00 | -3.01 |
| 19 | 0.71 | 0.50 | 4.00 | 0.08 | 0.20 | 3.80 | 0.46 | 0.38 | 1.18 | 0.46 | 1.75 | -0.58 |
| 20 | 0.5 | 0.42 | 1.41 | 0.42 | 1.41 | 0 | 0.47 | 0.48 | 2.05 | 0.54 | 1.10 | 0.95 |
| 21 | 0.42 | 0.33 | 0.96 | 0.50 | 4.00 | -3.05 | 0.47 | 0.74 | 0.64 | 1.00 | 4.00 | -3.36 |
| 22 | 0.54 | 0.50 | 4.00 | 0.42 | 1.41 | 2.59 | 0.52 | 0.46 | 1.75 | 0.54 | 0.10 | 1.65 |
| 23 | 0.54 | 0.50 | 4.00 | 0.25 | 0.68 | 3.33 | 0.50 | 0.56 | 0.15 | 0.56 | 0.15 | 0 |
| 24 | 0.54 | 0.38 | 1.18 | 0.31 | 0.88 | 0.30 | 0.33 | 0.33 | 0.96 | 0.67 | 0.44 | 0.52 |
| 25 | 0.58 | 0.77 | 0.74 | 0.62 | 0.31 | 0.43 | 0.54 | 0.5 | 4.00 | 0.42 | 1.41 | 2.6 |
| 26 | 0.50 | 0.08 | 0.20 | 0.08 | 0.20 | 0 | 0.43 | 0.36 | 1.08 | 0.50 | 4.00 | -2.92 |
| 27 | 0.58 | 0.92 | 1.41 | 0.77 | 0.74 | 0.67 | 0.50 | 0.42 | 1.41 | 0.42 | 1.41 | 0 |
| 28 | 0.50 | 0.46 | 1.75 | 0.46 | 1.75 | 0 | 0.54 | 0.38 | 1.18 | 0.31 | 0.88 | 0.30 |
| 29 | 0.54 | 0.50 | 4.00 | 0.42 | 1.41 | 2.59 | 0.58 | 0.77 | 0.74 | 0.62 | 0.31 | 0.43 |
| 30 | 0.58 | 0.31 | 0.88 | 0.15 | 0.39 | 0.50 | 0.67 | 0.67 | 0.44 | 0.33 | 0.96 | -0.52 |
| 31 | 0.50 | 0.62 | 0.31 | 0.62 | 0.31 | 0 | 0.54 | 0.67 | 0.44 | 0.58 | 0.20 | 0.24 |
| 32 | 0.50 | 0.42 | 1.41 | 0.42 | 1.41 | 0 | 0.52 | 0.94 | 1.56 | 0.90 | 1.28 | 0.28 |
| 33 | 0.33 | 0.33 | 0.96 | 0.67 | 0.44 | 0.51 | 0.50 | 0.42 | 1.41 | 0.42 | 1.41 | 0 |
| 34 | 0.54 | 0.67 | 0.44 | 0.58 | 0.20 | 0.24 | 0.58 | 0.92 | 1.41 | 0.77 | 0.74 | 0.67 |

Note:

"Correct percentage" means "mean correct response percentage”;

"hit rate" means "hit rate";

"Z of hit rate" means "Z score of hit rate";

"false rate" means "false rate";

"Z of false rate" means "Z score of false rate";

"d score" is equal to "Z score of hit rate minus Z score of false rate".
